# A Risk Score Model Based on Drug-Sensitivity-Related Genes Has the Potential to Predict Oral Squamous Cell Carcinoma Prognosis

**DOI:** 10.3290/j.ohpd.c_2124

**Published:** 2025-08-05

**Authors:** Yao Ma, Yunpeng Li, Sasa Ding, Peipei Sun

**Affiliations:** a Yao Ma Dentist, Department of Stomatology, Central Hospital Affiliated to Shandong First Medical University, Lixia District, Jinan 250000, Shandong Province, PR China. Conceived, designed and conducted the study; wrote the manuscript.; b Yunpeng Li Dentist, Department of Stomatology, Central Hospital Affiliated to Shandong First Medical University, Lixia District, Jinan 250000, Shandong Province, PR China. Conceived, designed and conducted the study; wrote the manuscript.; c Sasa Ding Dentist, Department of Stomatology, Central Hospital Affiliated to Shandong First Medical University, Lixia District, Jinan 250000, Shandong Province, PR China. Conceived, designed and conducted the study; wrote the manuscript.; d Peipei Sun Dentist, Department of Stomatology, Central Hospital Affiliated to Shandong First Medical University, Lixia District, Jinan 250000, Shandong Province, PR China. Conceived, designed and conducted the study; wrote the manuscript.

**Keywords:** OSCC, risk score, prognosis, IGF2BP2, PLAU, CEP55, CMYA5

## Abstract

**Objective:**

To develop a risk score model based on drug-sensitivity-related genes to predict the prognosis of patients with oral squamous cell carcinoma (OSCC).

**Methods and Materials:**

In this study, transcriptome from OSCC patients was downloaded from the Cancer Genome Atlas (TCGA) and International Cancer Genome Consortium (ICGC) databases, and differential gene expression analysis was performed using R’s ‘limma’ package. LASSO Cox regression identified key prognostic genes. We stratified patients into low- and high-risk groups and estimated survival rates using Kaplan-Meier. Gene set enrichment analysis (GSEA) and immune infiltration analysis were conducted to understand the potential pathways and tumour microenvironment. A nomogram model was constructed for prognosis prediction.

**Results:**

Our study identified 118 candidate genes from three data sets and narrowed them down to four prognostic genes (*IGF2BP2*, *PLAU*, *CEP55*, *CMYA5*) using univariate Cox regression and LASSO Cox regression. A risk score model was developed which could predict patient prognosis. The model’s prognostic value was independent of age, gender, and stage. A nomogram model incorporating risk score and age was constructed for personalised survival prediction. Tumour mutation burden analysis showed that the mutation rate of *TP53* was higher in the high-risk group. Immune landscape analysis uncovered distinct immune cell infiltration patterns and immune checkpoint expression levels between different risk groups, suggesting implications for immunotherapy strategies.

**Conclusion:**

The risk score model constructed using drug-sensitivity-related genes *IGF2BP2*, *PLAU*, *CEP55*, and *CMYA5* may predict the prognosis of OSCC patients.

Oral squamous cell carcinoma (OSCC) is a cancer that originates in the squamous cells lining the oral cavity, including the lips, tongue, gums, cheeks and throat. Squamous cell carcinoma is the most common type of oral cancer.^25^ OSCC is a significant global health concern, with the incidence and mortality ranking 16th in the world.^31^ It is more prevalent in older individuals, particularly those over 45 years of age, and it is more common in men than women.^1^ Risk factors encompass the use of tobacco products, excessive alcohol intake, and infection with the human papillomavirus (HPV).^43^ Moreover, increasing evidence indicates that periodontal diseases (such as periodontitis) are significantly associated with the development of OSCC.^34,47^ Periodontal pathogens such as *Porphyromonas gingivalis* and *Fusobacterium nucleatum* are associated with the cancerous state.^2,16^ The 5-year mortality rate for OSCC is close to 50% despite the medical development in recent decades.^9^ The prognosis for OSCC depends on factors such as the stage at diagnosis, the overall health of the patient, and the effectiveness of the chosen treatment. About half of OSCCs are diagnosed in advanced stages.^36^ Early detection, prognosis prediction, and treatment may generally result in better outcomes,^29^ and risk stratification plays a crucial role in guiding treatment decisions, allocating resources, and selecting patients for inclusion in clinical trials.^28^

Drug resistance in OSCC refers to the ability of cancer cells within the oral cavity to withstand the effects of therapeutic drugs, leading to a reduced or lost response to treatment. OSCC therapy faces a significant challenge with drug resistance, as this often leads to treatment failure, which in turn can cause the tumour to recur and potentially metastasise to other parts of the body. This phenomenon poses a significant challenge in the management of OSCC and other cancers, as it can restrict the efficacy of chemotherapeutic and targeted treatment approaches.^41^ Understanding the mechanisms and factors contributing to drug resistance is crucial for developing more effective treatment strategies. The development of drug resistance in OSCC is complex, involving processes such as the expulsion of drugs from cells, the transformation of epithelial cells into mesenchymal cells, the repair of DNA damage, and autophagy.^24^ In this research, we aimed to explore the drug sensitivity-related genes in OSCC patients to contribute to the treatment by providing information on drug sensitivity. Furthermore, because a single prognostic factor is insufficient for adequate risk stratification, there is growing interest in the development of multivariate prognostic models that quantitatively integrate two or more prognostic factors.^26^ We would try to construct a prognostic model for OSCC patients based on the identified drug-sensitivity-related genes.

## MATERIALS AND METHODS

### Research Objects

The mRNA expression profiles, along with corresponding clinical information, from 306 OSCC patients with complete survival details were obtained from TCGA database (https://tcga-data.nci.nih.gov/tcga/). Clinical details are available in Table 1, encompassing 307 tumour samples and 30 adjacent normal tissue samples. Additionally, mutation annotation format (maf) files for 311 OSCC patients were obtained from TCGA for subsequent analysis. OSCC data from the International Cancer Genome Consortium (ICGC) database were also downloaded for survival validation, comprising 40 samples with survival information. Datasets from the Gene Expression Omnibus (GEO) database (https://www.ncbi.nlm.nih.gov/geo/) were retrieved, including GSE42743 (from Affymetrix Human Genome U133 Plus 2.0 Array) and GSE75538 (from Illumina HumanHT-12 WG-DASL V4.0 R2 expression beadchip). GSE42743 consisted of 74 OSCC samples and 29 normal samples, while GSE75538 comprised 14 OSCC samples and 14 normal adjacent samples.

**Table 1:** ClinicopathologicalcharacteristicsofOSCCpatientsfromTCGAdatabase.

Characteristics	Patients (N = 306)
NO.	%	P value
Gender	Female	102	33.33%	0.7388
Male	204	66.67%
Age	≤61(Median)	157	51.31%	0.9791
>61(Median)	149	48.69%
Grade	GX	3	0.98%	0.8644
G1	49	16.01%
G2	191	62.42%
G3	62	20.26%
Unknown	1	0.33%
Survival Time	Long (>5years)	31	10.13%	0.4252
Short (<5years)	275	89.87%
OS status	Dead	143	46.73%	0.9479
Alive	163	53.27%
M	M0	289	94.44%	0.4619
M1	2	0.65%
Mx	12	3.92%
Unknown	3	0.98%
N	N0	160	52.29%	0.7329
N1	56	18.30%
N2	76	24.84%
N3	2	0.65%
Nx	9	2.94%
Unknown	3	0.98%
T	T1	18	5.88%	0.5887
T2	97	31.70%
T3	73	23.86%
T4	110	35.95%
Tx	5	1.63%
Unknown	3	0.98%
Primarysite	Anteriorfloorofmouth	2	0.65%	0.2139
Borderof tongue	1	0.33%
Cheekmucosa	19	6.21%
Floorof mouth	51	16.67%
Gum	8	2.61%
Hardpalate	4	1.31%
Lip	3	0.98%
Lowergum	2	0.65%
Mouth	20	6.54%
Overlappinglesionoflip, oral cavity and pharynx	69	22.55%
Palate	1	0.33%
Tongue	125	40.85%
Uppergum	1	0.33%


### Differential Gene Expression Analysis

All statistical analyses were done in the R language (4.2.0). For the analysis of differentially expressed genes (DEGs), the ‘limma’ function package in R language (version 3.52.0) 27 was applied with DEGs screened based on the criteria of |Log2FC| >2 and a P value <0.05.

### Functional Enrichment Analysis

Subsequently, we performed enrichment analysis including gene ontology (GO) terms (biological process (BP), molecular function (MF), and cellular component (CC)), and KEGG pathways for the obtained DEGs using the ‘clusterProfiler’ function package (version 4.7.1.002).^44^ Entries or pathways with a P value <0.05 were considered statistically significantly enriched.

### Culture of Cell Lines

In this study, two human cell lines were used: HOEC (BFN6072012669, BlueFBio Life Science, Shanghai, China), a normal human oral epithelial cell line, and SCC-4 (BNCC340434, BeNa Culture Collection, Beijing, China), an epithelial cell line extracted from the oral cavity of a 55-year-old male diagnosed with squamous cell carcinoma. HOEC cells were cultured in a high-glucose DMEM medium (PM150210, Procell Life Science & Technology Co, Wuhan, China) with the addition of 1% P/S (P1400, Procell, Wuhan, China) and 10% foetal bovine serum (FBS) (164210, Procell, Wuhan, China). SCC-4 cells were cultured in 90% DMEM-H/F12 (11330-032, Gibco (Grand Island Biological Company), Waltham, US) supplemented with 10% FBS, 400 ng/ml hydrocortisone (IH0100, Beijing Solarbio Science & Technology Co, Beijing, China). Cells were maintained at 37°C in a humidified incubator (MCO-18AC, Sanyo Electric (China) Co, Hong Kong, China) with 5% CO_2_.

### Quantitative Real-Time PCR (qRT-PCR)

Total RNA was isolated from cells using the TRNzol Universal reagent (DP424, Tiangen Biotech Co., Beijing, China). RNA integrity and concentration were determined using the Jenway Genova Nano UV spectrophotometer (Cole-Parmer, London, UK). Qualified RNA underwent reverse transcription with the StarScript III RT Mix (A230, Jiangsu Curovax Biotech Co., Suzhou, China). PCR was then conducted with the 2×RealStar Power SYBR Mix (A311, Suzhou, China) on a qPCR system (IQ5, Bio-Rad Laboratories, Hercules, US) according to the following thermal profile: initial denaturation at 95°C for 10 min, followed by 40 cycles of 95°C for 15 s and 60°C for 1 min. GAPDH served as the reference gene, with primer sequences detailed in Table 2. Triplicate reactions were run for each sample, and mRNA levels were quantified using the 2-ΔΔCT method.

**Table 2:** Primer sequences for RT-PCR

Genes	Forward Primer (5’-3’)	Reverse Primer (5’-3’)	Product
IGF2BP2	AAGCTAAGCGGGCATCAGTT	CGCAGCGGGAAATCAATCTG	176
PLAU	CCAAAATGCTGTGTGCTGCT	TTGTCCTTCAGGGCACATCC	145
GAPDH	GAAGGTGAAGGTCGGAGTC	GAAGATGGTGATGGGATTTC	172


### Least Absolute Shrinkage and Selection Operator (LASSO) Cox Regression Analysis

Univariate Cox regression analysis was performed on the samples, taking gene expression values into account, and the P value was used as a criterion to identify genes that are statistically significantly significantly associated with patient outcomes. Subsequently, the LASSO Cox regression method was applied to further narrow down the list of genes that have a meaningful impact on prognosis, utilising the ‘glmnet’ package (version 4.1-4).^6^

The genes that were ultimately selected were then used to compute an individual risk score for each sample, following a specific formula:


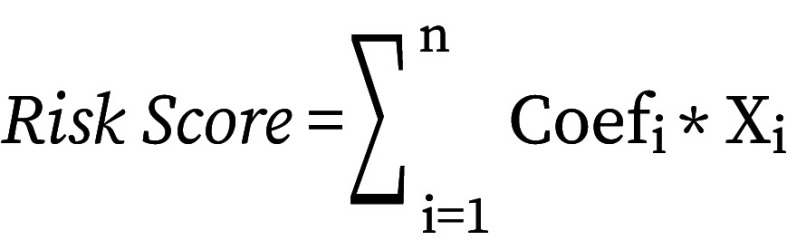


Here, Coef_i_ refers to the risk coefficient for each factor as calculated by the LASSO Cox model, while Xi represents the expression level of each factor, specifically the gene expression levels in this study.

### Survival Analysis

Patients were then stratified into OLRP (OSCC low-risk group) and OHRP (OSCC high-risk group) based on the median risk score. Using the R packages ‘survival’ (version 3.3-1) and ‘survminer’ (version 0.4.9), we estimated the overall survival rates for different groups based on the Kaplan–Meier (KM) method and assessed the significance of the differences in survival rates between groups using either the log-rank or Breslow tests. Additionally, we analysed whether the risk score could independently predict patient survival from other factors using a multivariate Cox regression model.

### Gene Set Enrichment Analysis (GSEA)

The R package ‘limma’ was utilised to perform differential expression analysis between OLRP and OHRP. The resulting DEGs were then subjected to GSEA using the R package ‘clusterProfiler’.^44^ KEGG pathways with a normalised enrichment score (NES) greater than 1 and a P value less than 0.05 were selected to identify statistically significantly enriched pathways.

### Immune Infiltration Analysis

CIBERSORT, a computational method for assessing the relative abundance of 22 different immune cell types within a cancer sample, was employed to calculate the immune cell proportions for each cancer specimen. The ‘estimate’ function from the corresponding package (version 1.0.13) was utilised to compute the immune scores for the samples.

### Nomogram Model for Prognosis Prediction

Nomograms have been extensively utilised for predicting cancer prognosis. To forecast the 1-, 3-, and 5-year survival probabilities for patients, a nomogram was constructed using the R language’s ‘rms’ package, based on the independent prognostic factors identified through multivariate Cox regression analysis. The calibration curve of the nomogram was also plotted to observe the relationship between the predicted probabilities and the actual incidence rates.

### Statistical Analyses

Comparisons between patients from different risk level groups were analysed using Chi-square tests or Fisher’s exact tests. Boxplots between two groups were generated using *t*-tests. A P value of less than 0.05 was considered to indicate statistical significance.

## RESULTS

### Candidate Gene Screening and Their Potential Functions

**Fig 1a to f:**
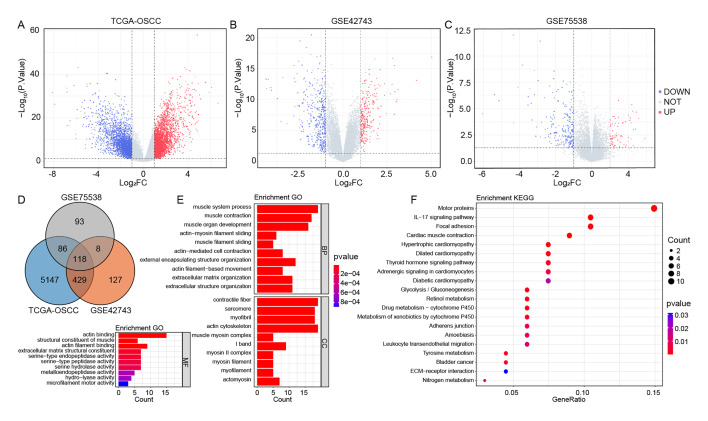
Candidate genes screening and their potential functions. (a to c) Volcano plots of differential expression analysis based on TCGA-OSCC, GSE42743, and GSE75538 datasets. (d) Intersection of DEGs from TCGA-OSCC, GSE42743, and GSE75538 data sets. (e) Top 10 significantly enriched GO terms of the three aspects on 118 candidate genes. (f) Top 20 significantly enriched KEGG pathways on 118 candidate genes.

We screened for DEGs between OSCC and normal groups based on three data sets. In the TCGA-OSCC data set, there were a total of 5780 DEGs, with 3063 upregulated genes and 2717 downregulated genes in OSCC samples compared to normal samples (Fig 1a, Table S1); in the GSE42743 data set, there were 682 DEGs, including 275 upregulated genes and 407 downregulated genes (Fig 1b, Table S1); in the GSE75538 data set, 305 DEGs were identified, among which 73 were upregulated and 232 were downregulated (Fig 1c, Table S1).

The intersection of the DEGs obtained from each data set yielded 118 shared genes (Fig 1d) as candidate genes. Subsequently, we performed GO and KEGG enrichment analyses on these 118 candidate genes. It was found that the candidate genes were statistically significantly enriched in GO terms such as ‘muscle system process’, ‘contractile fibre’, and ‘actin binding’, as well as in KEGG pathways like ‘motor proteins’. The top 10 most statistically significantly enriched GO terms are shown in Figure 1e, and the 20 significantly enriched KEGG pathways are shown in Figure 1f. Detailed results of the GO and KEGG enrichment analyses can be found in Table S2.

### Construction of a Prognostic Prediction Model for OSCC Patients

**Fig 2a to h:**
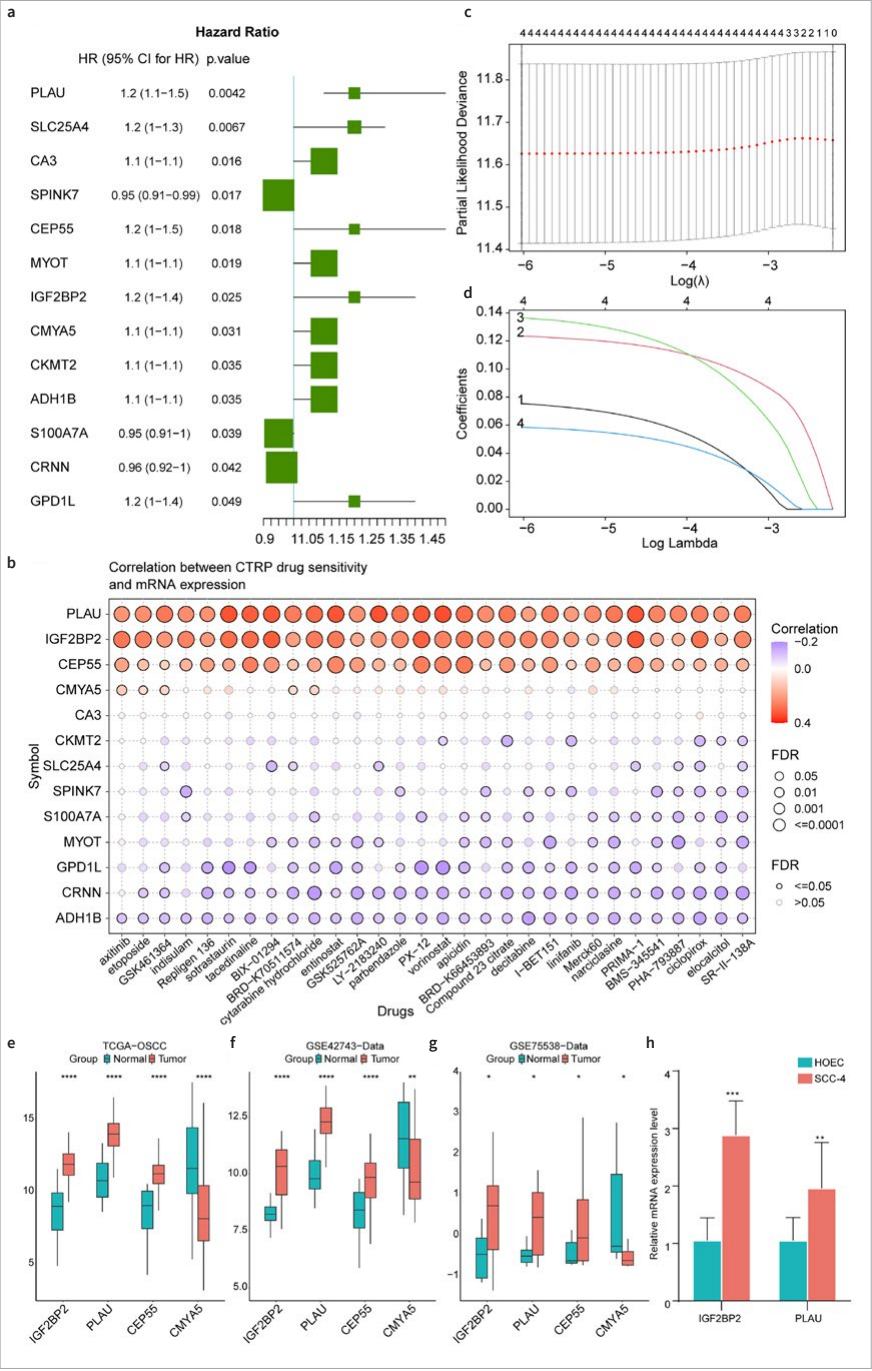
Construction of prognostic prediction model for OSCC patients. (a) Univariate Cox regression analysis of 13 genes. (b) Correlation between expression levels of 13 genes and drug sensitivity. (c and d) Determination of an optimal number of genes to be used in the model. (e to g) Expression levels of IGF2BP2, *PLAU*, *CEP55*, and *CMYA5* in TCGA-OSCC, GSE42743, and GSE75538 data sets. (h) Expression levels of IGF2BP2 and PLAU in qRT-PCR experiment. (* P <0.05; ** P <0.01; **** P <0.0001).

Based on the 118 candidate genes, we further narrowed down the number of target genes by univariate Cox regression analysis, and calculated the hazard ratio (HR) of each gene, resulting in 13 genes by the criteria of P <0.05 (Fig 2a). Subsequently, we analysed the drug sensitivity of 13 genes and identified the top four genes that were positively correlated with drug sensitivity (Fig 2b). We then conducted LASSO Cox regression analysis, and based on the lambda values corresponding to different numbers of genes in the LASSO Cox analysis, we determined the optimal number of genes to be four (Figs 2c and 2d, where the lambda value was the smallest). The four genes were *IGF2BP2*, *PLAU*, *CEP55*, and *CMYA5*. We analysed the expression differences of these four genes between tumour patients and normal samples in the TCGA-OSCC data set (Fig 2e), *GSE42743* (Fig 2f), and *GSE75538* (Fig 2 g) data sets. The results showed that *IGF2BP2*, *PLAU*, and *CEP55* were found to be statistically significantly higher expressed in tumours than in normal samples, while *CMYA5* was statistically significantly lower expressed in tumours. The higher expression levels in the tumour of *IGF2BP2* and *PLAU* were validated by qRT-PCR (Fig 2h).

Genes’ expression levels were then weighted according to the regression coefficients from the LASSO Cox regression analysis to establish a risk score model for predicting patient prognosis. Twhe risk score was calculated as follows: risk score = (*IGF2BP2* × 0.07528122) + (PLAU × 0.12344856) + (CEP55 × 0.13650156) + (CMYA5 × 0.05853046). We calculated the risk score for each patient and regrouped the samples in the TCGA data set and ICGC validation set into OLRP and OHRP.

### Validation of the Risk Score Model for OSCC Patients

**Fig 3:**
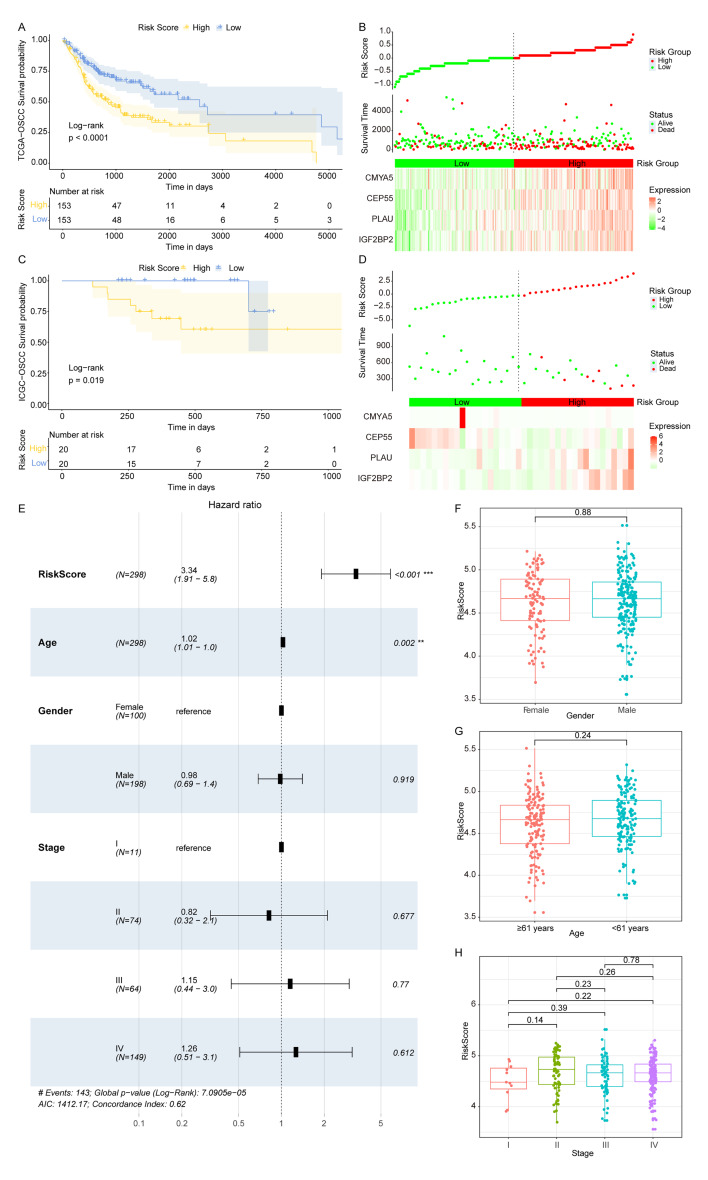
Validation of the risk score model for OSCC patients. (a) KM curves between OLRP and OHRP based on TCGA-OSCC data set. (b) Prognostic feature distribution map, patient survival map and heatmap of IGF2BP2, *PLAU*, *CEP55*, and *CMYA5*’s expression levels based on TGCA-OSCC data set. (c) KM curves between OLRP and OHRP based on the validated data set ICGC-meta. (d) Prognostic feature distribution map, patient survival map and heatmap of *IGF2BP2*, PLAU, *CEP55*, and CMYA5’s expression levels based on ICGC-meta dataset. (f to h) Risk score in OSCC samples of different genders, different ages, and different stages, respectively (** P <0.01, *** P <0.001).

To further elucidate the prognostic value of the risk score, we conducted additional analyses across various clinical and pathological parameters of OSCC patients. In all TCGA-OSCC patients, a higher risk score was associated with a lower survival rate, and the KM curves showed a statistically significantly difference between OLRP and OHRP (Fig 3a). Additionally, the prognostic feature distribution map displayed that risk score and the expression of *IGF2BP2*, *PLAU*, *CEP55*, and *CMYA5* were correlated with lower patient survival (Fig 3b). Simultaneously, the results from the validation set ICGC-meta indicated a statistically significantly higher survival rates in OLRP compared to OHRP (Fig 3c). The similar pattern of risk score, gene expression, and patient dead or alive status was also detected in this validation data set (Fig 3d).

We included age, gender, stage, and risk score as four factors in a multivariate Cox regression analysis to determine if the risk score is an independent prognostic indicator. The results showed that the risk score and age were statistically significantly associated with overall survival, with OLRP samples having a lower risk of death, making it a reliable prognostic factor (HR = 3.34, 95% CI: 1.91–5.8, P <0.001) (Fig 3e). Furthermore, no statistically significant differences in risk score were found between samples of different genders (Fig 3f), samples from patients ≤61 years old and those >61 years old (Fig 3 g), or samples from different stages (Fig 3h), indicating that the risk score could serve as an independent indicator to predict the prognosis of patients with OSCC.

### Nomogram Model Based on Risk Score and Age for OSCC Prognostic Prediction

**Fig 4:**
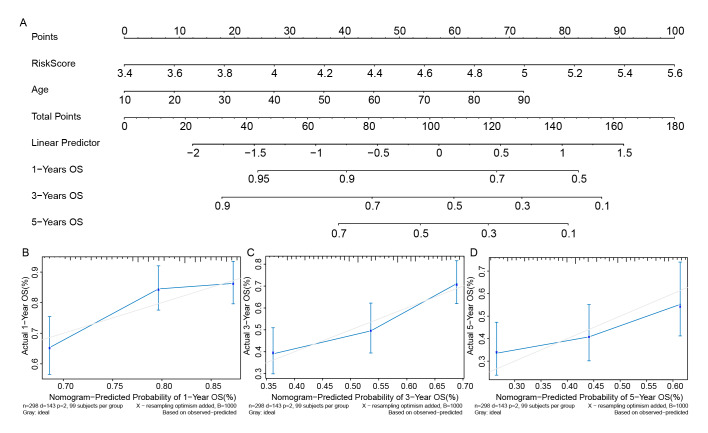
Nomogram model based on risk score and age for OSCC prognostic prediction. (a) A nomo-gram constructed by risk score and age to facilitate personalised risk assessment for OSCC patients. (b) The calibration curve of the nomogram for the 1-year, 3-year and 5-year OS predictions for OSCC patients.

Next, building upon the identification of the risk score and age as independent prognostic factors, we constructed a nomogram model (Fig 4a) to facilitate personalized risk assessment by estimating overall survival (OS) for each patient. For each individual, two vertical lines are drawn to determi-ne the points assigned to each factor in the nomogram. The total score, indicated on the ‘Total Points’ axis, is then used to project a horizontal line that corresponds to the probabilities of 1-year, 3-year, and 5-year overall survival in OSCC patients. The calibration curves for the 1-, 3-, and 5-year OS predictions closely approximate the ideal reference line (a 45-degree line through the origin), indicating strong agreement between the model’s predictions and actual outcomes over these time intervals (Figs 4b–4d).

### Potential Pathways Functioning Differentially Between OSCC Samples with High- and Low-Risk Scores

**Fig 5:**
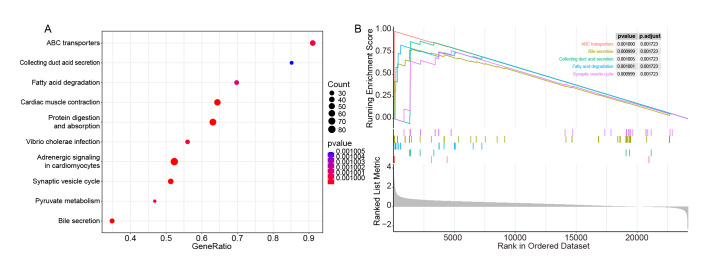
Potential pathways functioning differentially between OSCC samples with high- and low-risk scores. (a) Top 10 enriched KEGG pathways between OLRP and OHRP by GSEA analysis. (b) GSEA analysis between OLRP and OHRP.

To explore potential pathways functioning differentially between OSCC samples with high- and low-risk scores, we conducted GSEA between OLRP and OHRP groups based on KEGG pathways. The results showed that 128 KEGG pathways were statistically significantly enriched with a threshold of |NES| >1 and P value <0.05. Among these, the top ten KEGG pathways with the lowest P values were visualised (Fig 5a) (Table S3). Pathways such as bile secretion, synaptic vesicle cycle, ABC transporters, fatty acid degradation, and collecting duct acid secretion were found to be statistically significantly activated (Fig 5b).

### The Tumour Mutation Burden (TMB) In OSCC Samples with High- and Low-Risk Scores

**Fig 6:**
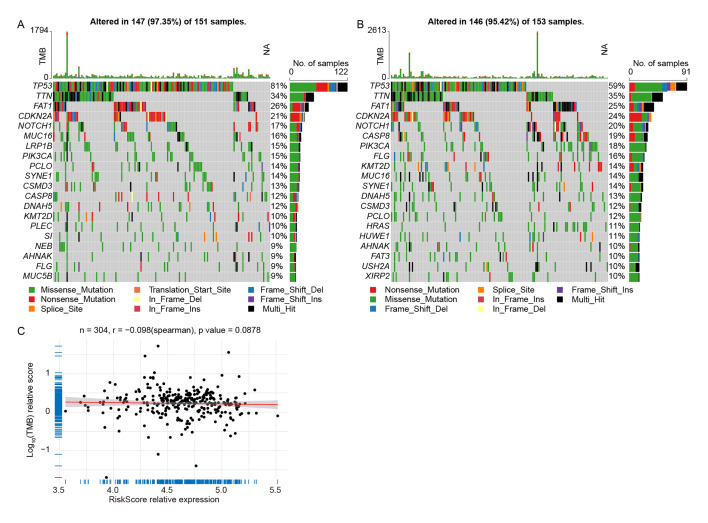
The tumour mutation burden (TMB) in OSCC with high- and low-risk scores. (a and b) TMB analysis in OHRP and OLRP. (c) Correlation between TMB and risk score.

The TMB reflects the cumulative extent of mutations in tumour cells and is closely associated with the biological characteristics of cancer and has been recognised as a crucial factor influencing the prognosis and treatment outcomes of cancer patients.^30^ By comparing the differences in TMB between OHRP and OLRP, we can explore tumour biology features relevant to risk groups, aiding in a deeper understanding of the mechanisms underlying OSCC development. We observed differences in somatic mutations in the TCGA-OSCC cohort, calculating TMB of OHRP and OLRP samples. The mutation analysis revealed that the mutation rate of *TP53* gene was 81% in OHRP (Fig 6a), while in OLRP, its mutation rate was 59% (Fig 6b). The TMB and risk score results indicated a negative correlation between TMB and risk score (Fig 6c).

### Immune Landscape Analysis of High- and Low-Risk Score OSCC Samples

**Fig 7a to c:**
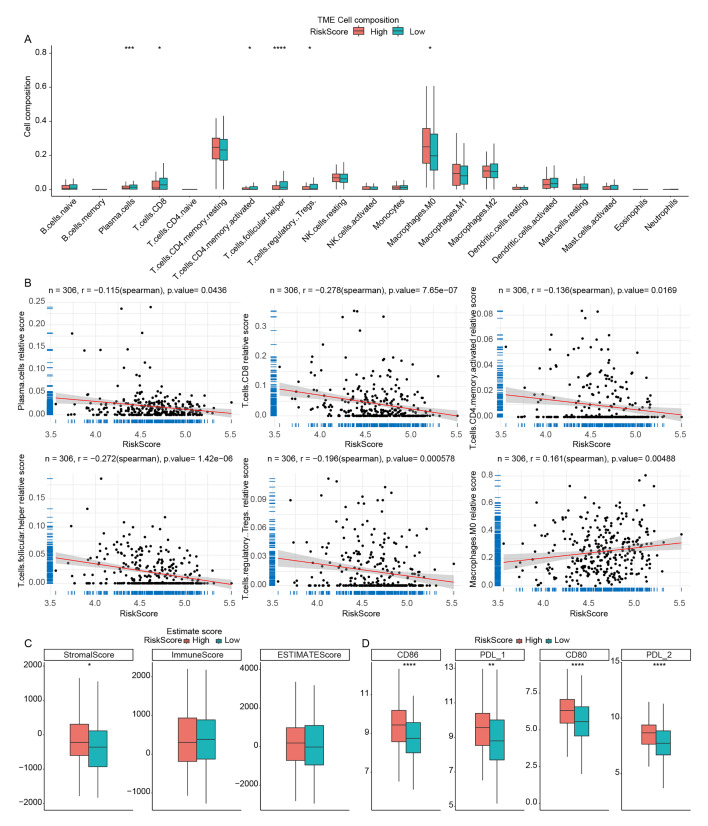
Immune landscape analysis of high- and low-risk score OSCC samples. (a) Infiltration proportion of different types of immune cells in OHRP and OLRP samples. (b) Correlation between immune cells infiltration levels and risk score. (c) StromalScore, ImmuneScore, and ESTIMATEScore in OHRP and OLRP. (d) Expression levels of immune checkpoint genes in OHRP and OLRP (* P <0.05; ** P <0.01; *** P <0.001; **** P <0.0001).

The tumour microenvironment (TME) is also a key factor in drug resistance.^5^ Researching infiltration proportions of different immune cell types between high- and low-risk groups is essential to understanding the impact of risk stratification on the TME and immune response. Investigating these differences can provide insights into the potential correlation between immune cell infiltration patterns and the level of risk in OSCC patients. Using the CIBERSORT algorithm, we analysed the differences in immune cell infiltration among the 22 immune cell types between OLRP and OHRP samples. The results showed statistically significant differences in the infiltration proportions of six immune cell types between OLRP and OHRP samples. The infiltration proportion of Macrophages M0 was statistically significantly higher in OHRP, while plasma cells, CD8 T cells, activated CD4 memory T cells, follicular helper T cells, and Tregs were statistically significantly lower in OHRP than OLRP (Fig 7a). Furthermore, through correlation analysis, the risk score was found to be significantly negatively correlated with plasma cells, CD8 T cells, activated CD4 memory T cells, follicular helper T cells, and Tregs, and significantly positively correlated with macrophages M0 (Fig 7b). The stromal score was statistically significantly higher in OHRP compared to OLRP samples (Fig 7c).

Immune checkpoint genes play a pivotal role in regulating the immune response, and their expression levels can impact the effectiveness of immunotherapy. Studying these genes in different risk groups may help identify potential differences in immune evasion mechanisms and can guide the development of personalised treatment strategies. In this study, expression analysis of nine immune checkpoint genes (*PD-1 (PDCD1)*, *CTLA4*, *PD-L1 (CD274)*, *PD-L2 (PDCD1LG2)*, *CD86*, *LAG3*, *TIGIT*, *CD80*) between OLRP and OHRP revealed significant differences in the expression of four immune checkpoint genes (*PD-L1 (CD274)*, *PD-L2 (PDCD1LG2)*, *CD80*, *CD86*), with OHRP samples showing significantly higher expression than OLRP samples (Fig 7d).

## DISCUSSION

Substantial progress has been made in biological research, as well as in clinical diagnostics and treatments, in recent years.^13,15^ However, the development of resistance to clinical drugs continues to pose a significant obstacle to the success of therapies for OSCC. The existing staging system for OSCC has certain limitations, primarily because it focuses mainly on the characteristics of the disease itself, neglecting other factors that influence prognosis. There is a pressing need for more personalised prognostic models that incorporate a broader range of variables.^45^ In this research, a prognostic model for OSCC patients was established by use of drug-sensitivity-related genes *IGF2BP2*, *PLAU*, *CEP55*, and *CMYA5*, where high-risk scores indicated poor prognosis.

IGF2BP2, or insulin-like growth factor 2 mRNA binding protein 2, is an RNA-binding protein that plays a crucial role in post-transcriptional regulation, influencing mRNA stability and translation,^40^ and has been implicated in various physiological processes and diseases, including OSCC. Proteins encoded by IGF2BP play a substantial role in the metabolic processes of tumours, with particular emphasis on their involvement in the metabolic pathways of head and neck squamous cell carcinoma (HNSCC).^39^ Corresponding to our research, *IGF2BP2* has been found to be upregulated in OSCC promoting OSCC progression in many other studies. The proliferation, invasion, and migration of OSCC cells can be inhibited by knockdown of IGF2BP2.^46^ Loss of Igf2bp2 leads to increased release of inflammatory cytokines, thereby exacerbating periodontitis.^22^ Periodontitis was suspected to be associated with OSCC.^10^ Moreover, this gene has been identified as a prognostic marker in OSCC associated with immunity and OSCC genesis,^35^ and its expression is able to upregulate EREG resulting in OSCC cell invasion and epithelial-mesenchymal transition (EMT) promotion.^19^ In laryngeal squamous cell carcinoma (LSCC), *IGF2BP2* can regulate the modification of N6-methyladenosine (m6A) which is a key factor in LSCC genesis. Overexpressed *IGF2BP2* in LSCC has been found to promote LSCC cell proliferation and invasion *in vitro*.^33^

The *PLAU* gene, encoding the urokinase-type plasminogen activator (uPA), is a crucial genetic element involved in various physiological processes, including fibrinolysis, tissue remodelling, and cell migration.^11^ It promotes the development of many cancers, such as cholangiocarcinoma,^12^ cervical cancer,^8^ and OSCC. In LSCC, stability of PLAU is regulated by a m6A methyltransferase WTAP resulting in cancer progression.^20^ As same as *IGF2BP2*, PLAU can also promotes cell proliferation and EMT in HNSCC that PLAU silencing inhibits the migration of OSCC Stage 4. It has been identified as an independent prognostic marker for HNSCC patients with higher expression level representing poorer prognosis. The expression level of PLAU is negatively correlated with CD4 T cell and Tregs.^18^ The risk score in our study also negatively associates with CD4 T cell and Tregs.

CEP55 (centrosomal protein 55) is essential for cell division and cytokinesis, playing a key role in regulating centrosome dynamics and cell cycle progression.^17^ It is often expressed lowly in most normal human tissues.^7^ Its overexpression causes genomic instability, which is a characteristic of cancer, such as breast cancer,^14^ oesophageal squamous cell carcinoma,^42^ etc. High expression level of CEP55 is related to poor prognosis of OSCC patients, independent of pathological stages or grades (32 as our risk score). As with *IGF2BP2* and *PLAU*, *CEP55* has been identified as a marker for HNSCC as well.^37^ CMYA5, namely cardiomyopathy-associated 5, is a gene associated with cardiomyopathies.^21^ CMYA5 has been detected as a DEG between lymph node-positive and negative OSCC patients.^23^ The role *CMYA5* plays in OSCC requires further research.

Patients with elevated tumor mutational burden (TMB) exhibit higher response rates to immune checkpoint inhibitor (ICI) therapy compared to those with lower TMB levels in the context of cancer treatment. A prognostic model based on TMB-related genes has the potential to predict OSCC prognosis and effectively stratify patients.^38^ Cigarette smoking has been investigated to associate with an elevated occurrence of *TP53* mutations, resulting in the inactivation of *TP53* and an augmented likelihood of developing oral tumours,^3^ which is corresponding to our research that the mutation rate of TP53 is higher in OHRP. The expression levels of immune checkpoints in OHRP are significantly higher than in OLRP, suggesting higher response rates to ICI therapy.

While this study has developed a personalised prognostic model for OSCC, several limitations must be acknowledged. Firstly, the reliance on public data from TCGA and ICGC databases may introduce biases that are not accounted for. Secondly, the generalizability of our findings to diverse populations is uncertain, as the cohorts used in this study may not represent the global demographic distribution. Thirdly, the prognostic model, although validated, requires further validation in prospective clinical trials to confirm its predictive accuracy and utility in real-world settings. Additionally, the mechanisms underlying the identified genes’ roles in OSCC progression and drug resistance are not fully elucidated and warrant further mechanistic studies.

## CONCLUSIONS

In conclusion, this study has developed a prognostic model for OSCC patients by integrating drug sensitivity-related genes *IGF2BP2*, *PLAU*, *CEP55*, and *CMYA5*. The model, which assigns a risk score to patients, has demonstrated the ability to separate patients into high- and low-risk groups, with a higher risk score correlating with a poorer prognosis. The identified genes play significant roles in tumour progression and have been associated with various cancer-related processes. The immune landscape analysis has also provided insights into the differential immune cell infiltration patterns between high- and low-risk OSCC samples, which could be crucial for understanding the tumour microenvironment and developing targeted immunotherapies. The nomogram model constructed in this study offers a personalised approach to predicting survival probabilities and could serve as a tool in clinical decision-making for OSCC treatment. Overall, this research contributes a step to the advancement of personalised medicine in OSCC by providing a comprehensive prognostic model that considers both genetic and immunological factors.

### Declarations

#### Availability of data and materials

The data set analysed during the current study was downloaded from the TCGA database (https://tcga-data.nci.nih.gov/tcga/), the ICGC and the Gene Expression Omnibus (GEO) database (https://www.ncbi.nlm.nih.gov/geo/).

### Supplement Tables

**Table S1** Differentially expressed genes between OSCC and normal groups in the TCGA, GSE42743, and GSE75538 data sets

https://www.quintessence-publishing.com/quintessenz/journals/articles/downloads/ohpd_2025_7596_ma_table_s1.xls

**Table S2** Detailed results of GO and KEGG enrichment analyse

https://www.quintessence-publishing.com/quintessenz/journals/articles/downloads/ohpd_2025_7596_ma_table_s2.xls

**Table S3** Detailed results of GSEA analysis

https://www.quintessence-publishing.com/quintessenz/journals/articles/downloads/ohpd_2025_7596_ma_table_s3.xls
